# Step by step management in neurospine trauma

**Published:** 2012-11

**Authors:** Hamid Reza Saeidiborojeni, Pezhman Soliemani

**Affiliations:** ^*a*^Department of Neurosurgery , Imam Reza Hospital, Kermanshah University of Medical Sciences, Kermanshah, Iran.; ^*b*^Department of Neurosurgery, Taleghani Hospital, Kermanshah University of Medical Sciences, Kermanshah, Iran.

## Abstract

**Background:**

Nowadays, the question of separation between training and clinics in many clinical fields is extensively debated, and all universities endeavor to present their theoretical education in a close connection with the clinics’ settings. Medical students require novel educational approaches that will enable them to have an efficient performance in clinical settings. The present study was aimed at evaluating the efficacy of algorithmic and lecture-based training on learning of interns.

**Methods:**

In this experimental study, we assessed scores obtained by two groups of interns (case and control groups) that each group consisted of 30 interns as, on a multiple-choice questionnaire basis with high level of validity and reliability. The scores of interns for prior the training were compared with those scores obtained for two weeks post-training. The scores for case and control group were presented using the algorithmic and lecture-based method, respectively. Data were analyzed using the SPSS software (version 15). The independent t-test, paired t-test and ANOVA assessments were used for analyses.

**Results:**

In the case group, the mean scores of interns increased from 10.034 (SD= 1.56) prior the training to 15.23 (SD=1.57) after algorithmic training, indicating a significant difference. In the control group, the mean scores of interns increased from 10.47 (SD=2.43) before training to 12.33 (SD=1.54) after lecture-based training, indicating a significant difference. Analysis of variance indicated that the mean score of interns after training in the case group was significantly higher, compared to those of the control group.

**Conclusions:**

Our findings demonstrate that training improves learning, and as medical students are more active in clinical fields, using novel methods of education such as algorithmic training may be more efficient compared to other methods.

**Keywords:**

Training, Algorithmic Training, Medical Students, Medicine


					Volume 4, Suppl. 1 Nov 2012
Publisher: Kermanshah University of Medical Sciences
URL: http://www.jivresearch.org
Copyright:
Congress of Iranian Neurosurgeons, 14-16 November 2012, Kermanshah, Iran
Paper No. 10
Step by step management in neurospine trauma
Hamid Reza Saeidiborojeni a,*
, Pezhman Soliemani b
a
Department of Neurosurgery , Imam Reza Hospital, Kermanshah University of Medical Sciences, Kermanshah, Iran.
b
Department of Neurosurgery, Taleghani Hospital, Kermanshah University of Medical Sciences, Kermanshah, Iran.
Abstract:
Background: Nowadays, the question of separation between training and clinics in many clinical fields is extensively
debated, and all universities endeavor to present their theoretical education in a close connection with the clinics'
settings. Medical students require novel educational approaches that will enable them to have an efficient
performance in clinical settings. The present study was aimed at evaluating the efficacy of algorithmic and lecture-
based training on learning of interns.
Methods: In this experimental study, we assessed scores obtained by two groups of interns (case and control groups)
that each group consisted of 30 interns as, on a multiple-choice questionnaire basis with high level of validity and
reliability. The scores of interns for prior the training were compared with those scores obtained for two weeks post-
training. The scores for case and control group were presented using the algorithmic and lecture-based method,
respectively. Data were analyzed using the SPSS software (version 15). The independent t-test, paired t-test and
ANOVA assessments were used for analyses.
Results: In the case group, the mean scores of interns increased from 10.034 (SD= 1.56) prior the training to 15.23
(SD=1.57) after algorithmic training, indicating a significant difference. In the control group, the mean scores of
interns increased from 10.47 (SD=2.43) before training to 12.33 (SD=1.54) after lecture-based training, indicating
a significant difference. Analysis of variance indicated that the mean score of interns after training in the case group
was significantly higher, compared to those of the control group.
Conclusions: Our findings demonstrate that training improves learning, and as medical students are more active in
clinical fields, using novel methods of education such as algorithmic training may be more efficient compared to other
methods.
Keywords:
Training, Algorithmic Training, Medical Students, Medicine
*Corresponding Author at:
Hamid Reza Saeidiborojeni: Associate professor, Neurosurgery Department, Kermanshah University of Medical Sciences, Kermanshah, Iran.
Tel: 98-918-131-1068, Email: dr.hamidrezasaeidi@yahoo.com, (Saeidiborojeni H.).

				

